# Direct synthesis of highly stretchable ceramic nanofibrous aerogels via 3D reaction electrospinning

**DOI:** 10.1038/s41467-022-30435-z

**Published:** 2022-05-12

**Authors:** Xiaota Cheng, Yi-Tao Liu, Yang Si, Jianyong Yu, Bin Ding

**Affiliations:** Innovation Center for Textile Science and Technology, College of Textiles, Donghua University, Shanghai, 201620 China

**Keywords:** Synthesis and processing, Porous materials

## Abstract

Ceramic aerogels are attractive for many applications due to their ultralow density, high porosity, and multifunctionality but are limited by the typical trade-off relationship between mechanical properties and thermal stability when used in extreme environments. In this work, we design and synthesize ceramic nanofibrous aerogels with three-dimensional (3D) interwoven crimped-nanofibre structures that endow the aerogels with superior mechanical performances and high thermal stability. These ceramic aerogels are synthesized by a direct and facile route, 3D reaction electrospinning. They display robust structural stability with structure-derived mechanical ultra-stretchability up to 100% tensile strain and superior restoring capacity up to 40% tensile strain, 95% bending strain and 60% compressive strain, high thermal stability from −196 to 1400 °C, repeatable stretchability at working temperatures up to 1300 °C, and a low thermal conductivity of 0.0228 W m^−1^ K^−1^ in air. This work would enable the innovative design of high-performance ceramic aerogels for various applications.

## Abstract (web summary)

Ceramic aerogels are generally brittle and often tend to structurally collapse under large external tensile strain. Here the authors synthesize large-scale stretchable ceramic aerogels with interwoven crimped nanofibers by combining electrohydrodynamic method and 3D reaction electrospinning.

## Introduction

Ultralight ceramic aerogels have been widely applied for thermal, electrical, magnetic, medical, optical and chemical applications owing to their low density and thermal conductivity, high specific surface area, high porosity, and chemical and thermal inertness^1–4^. However, conventional ceramic aerogels with typical pearl necklace-like structures are brittle and often tend to structurally collapse under large external stresses or strains caused by inefficient structural continuity and connection^5–8^. Prior efforts to improve mechanical properties of ceramic aerogels by manufacturing ceramic aerogel materials with continuous structural units^9–13^, such as nanofibre or nanosheet. Due to the absence of necklace-like structures and defects, these continuous elements could yield additional degrees of freedom to avoid brittle failure of building blocks for improving compressive brittleness of ceramic aerogels, but cannot address the stretchable brittleness. When used as the main medium for protecting the human body and equipment, the poor stretchable properties of ceramic aerogels have resulted in limited applications in extreme environments such as aerospace and defense.

As a prerequisite for their efficient application, ceramic aerogels must exhibit promised mechanical properties including both stretchability, compressibility and flexibility. Owing to their relatively simple and straight building blocks, fragile connections or lamellar structures^14–17^, all existing aerogel-like 3D materials undergo transient structural fracture or slipping under even small tensile strain, and they are far from meeting the needs of composite processing and practical applications, for instance, boron nitride ceramic aerogels prepared by chemical vapour deposition^1^, ceramic aerogels developed by the freeze-shaping method (silica nanofibrous aerogels^14,15^, mullite-based nanofibrous aerogels^18^, Al_2_O_3_ nanorod aerogels^19^ and SiC@SiO_2_ nanowire aerogels^20^) and lamellar ceramic fibrous aerogels directly obtained by spinning^21,22^. In addition, these methods generally have the problems of complicated preparation processes, high cost and difficult mass production. Alternatively, a natural luffa stem with crimped and entangled structures has provided us material design inspirations for improving the stretchability and structural stability of ceramic aerogels. When subjected to tensile stress or strain, crimped structures can transform stretching into unbending of the structures, and dense entanglements are conducive to ensuring that tension transmits along the element and to many other elements, effectively avoiding stress concentration^23–26^. However, ceramics are more difficult to process than metals and polymers, especially into complex shapes^27^, and thus, improvement in stretchable properties of ceramic aerogels via structural design being a major unresolved challenge. Therefore, exploiting a simple, rapid, and low-cost mass-production strategy for the preparation of ceramic nanofibres with complex shapes is the key to making a breakthrough in ceramic aerogels with stretchable properties.

Here, we show a electrohydrodynamic methodology, 3D reaction electrospinning, to directly manufacture ceramic nanofibrous aerogels with interwoven crimped-nanofibre structures. The 3D interwoven crimped-nanofibre structured ceramic aerogels (ICCAs) exhibit superior performance such as ultralight weight, flexibility, stretchability, compressibility, fatigue tolerance, and ultralow thermal conductivity. The ICCAs can be stretched from their original morphology to 100% tensile strain without fracture and simultaneously exhibit superior restoring capacity in response to large deformations of more than 40% tensile, 60% compressive or 90% bucking strain, as well as robust fatigue-tolerance for 100,000 cycles. Moreover, the mullite phase provides the ceramic aerogel with thermal stability from -196 to 1400 °C, and the aerogels still have repeatable stretchability after calcination at 1300 °C for 1 h. Finally, we demonstrate that ICCAs can be applied as high-performance thermal insulation materials. Meanwhile, we prepare a large scale nanofibrous aerogel with 170 cm long, 130 cm wide, and 12 cm high via the method. These results have broad technological and engineering implications for personal protective equipment, thermal protection systems in space vehicles and flexible wearable electronics.

## Results

### 3D reaction electrospinning of ceramic nanofibrous aerogels

We designed and fabricated a ceramic nanofibre aerogel with an interwoven crimped-nanofibre structure by controlling the coagulation rate of the jet during electrospinning. In conventional electrospinning, a charged fluid jet is formed in a conical droplet by an electrostatic force and accelerates towards the collector with stretching slenderization. Many investigations have indicated that the travelling direction of charged fluid jets lacking dampening viscoelastic forces change from the centerline under the action of the applied electric potential during electrospinning^28,29^, inducing the onset of bending instability and the formation of a crimped-fibre structure. Ultimately, the crimped-fibre structure collapses or is straightened under the action of surface tension and electrostatic forces, and randomly oriented 2D fibrous membranes are formed by deposition of the unsolidified jet^30^. Creating 3D nanofibrous structures by electrospinning has been intractably difficult thus far. To directly assemble ceramic fibrous aerogels with interwoven crimped-nanofibre structures, we designed a 3D reaction electrospinning method controlled by a sol-gel reaction in the jet flow. The rate of gelation of the sol jet was controlled to achieve precise control of the jet shape within milliseconds by tailoring the extent of protonation of colloidal particles.

**Fig. 1:**
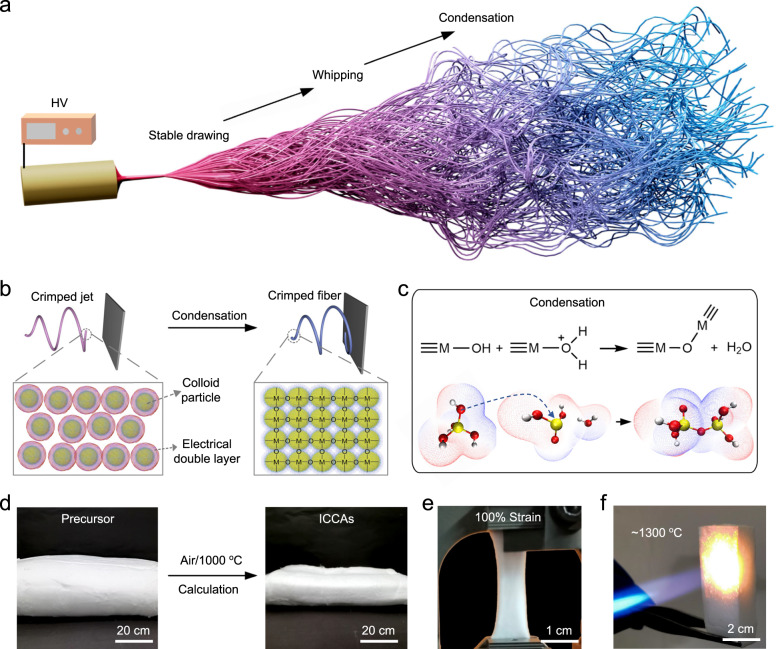
Design and fabrication of ICCAs. **a**–**c** Illustration of 3D reaction electrospinning for directly fabricating ceramic nanofibrous aerogels. **d** Images of precursor aerogels and ICCAs. **e**–**f** ICCAs stretched from their original morphology up to 100% strain without any fracture and heated by a butane blowtorch.

Figure 1a–c shows the 3D reaction electrospinning process, which was performed using a sol solution with high conductivity (12910 μs/cm) and low viscosity (18.76 cp). Our previous work suggested that excess charge density of the liquid would result in the formation of spherical droplets instead of cylindrical jets^31,32^. More excitingly, we changed the ejection mode of the conical droplet to a multijet mode (Supplementary Movie 1) by adding low amounts (0.1 wt%) of high-molecular-weight polymers while the condensation between colloidal particles was not impacted. The speed of a single spinneret hole in this method was 5~10 times higher than that in the fabrication of ceramic nanofibres by conventional electrospinning^33^, enabling industrial-scale production. The stability of the jet noticeably decreased, and the jet experienced whipping instability after being elongated into a slender straight jet due to the high surface potential, forming a 3D crimped structure (Supplementary Fig. 1). With rapid solvent evaporation, the distance between colloidal particles and the repulsive coulombic interaction caused by the electrical double layer was reduced, resulting in the aggregation of colloidal particles^34–36^. Subsequently, the highly reactive colloidal particles tend to form highly cross-linked and robust skeletons via condensation and jets solidified (Fig. 1b), suppressing the deformation and collapse of the 3D crimped nanofibrous structures. We proved that the distance between the protonated oxygen atom of ≡M-OH and the metal atom increased, creating a good leaving group (Fig. 1c and Supplementary Table 1). In addition, the central metal atom of the protonated group became more electrophilic due to the withdrawal of electron density. The electrophilic properties of the central metal atom rendered it more susceptible to attack by nucleophilic groups, indicating that protonation provided a more reactive site for condensation. In contrast, beaded nanofibres formed when the extent of protonation was too low, while microfibres formed when the extent of protonation was too high (Supplementary Fig. 2).

To enhance the reaction rate for condensation of colloidal particles, we produced a sol spinning solution with both high gelation reactivity during the spinning process and stability before spinning by two-step acid catalytic processes. First, colloidal particles with low condensation reactivity were prepared by hydrolyzing a metal alkoxide under a low concentration of H^+^. Subsequently, we acidified the sol solution to a lower pH to increase the extent of protonation of colloidal particles by dropwise addition of ethanol-diluted hydrochloric acid solution. The final sol solution exhibited stability and spinnability even after one week, as an electrical double layer was formed around the colloidal particles again and the repulsive barrier was increased, which was proven by the zeta potential (Supplementary Fig. 3). To gain deeper insight into the different spinning processes of three sol spinning solutions with different extents of protonation, we obtained Fourier transform infrared (FTIR) spectra of untreated precursor nanofibrous aerogels and characterized the particle sizes of untreated aerogels dissolved in water via dynamic light scattering (DLS). The condensation reaction occurred during the spinning process after the extent of protonation increased, as confirmed by the new bands near 813 cm^−1^ and 1090 cm^−1^ in the FTIR spectra^37^ (Supplementary Fig. 4) and the increase in the particle size from 8 nm to 140 nm (Supplementary Fig. 5). Moreover, the untreated aerogels fabricated with the least reactive sol could promptly dissolve in water, and the solution was transparent, indicating that no reaction occurred between the low-reactivity colloidal particles in the spinning process. The other two aerogels obtained from a more reactive sol were insoluble in water and the dispersion solution exhibited white haze (Supplementary Fig. 6), which further proved that the reaction occurred during the spinning process. Subsequently, we interknitted the crimped nanofibre to obtain a 3D interwoven crimped-fibre structured nanofibrous aerogel precursor by tailoring the vertical movement of the spinning nozzle and the collector. Finally, the aerogel precursors were calcined at 1000 °C for 1 h in air, and the ICCAs were obtained (Fig. 1d). The final ceramic nanofibrous aerogels exhibited thermal stability and stretchability. The integral ceramic nature of the ICCAs allows them to withstand even high-temperature flames 1300 °C (butane blowlamp)^20,38^ without any damage or deformation (Fig. 1e) and be stretched from their original morphology to 100% tensile strain without fracture (Fig. 1f, Supplementary Fig. 7 and Supplementary Movie 2). More excitingly, using different highly reactive sol spinning solutions, we fabricated various ceramic nanofibrous aerogels, such as mullite, Al_2_O_3_, ZrO_2_ and Al_2_O_3_-ZrO_2_ (Supplementary Fig. 8).

### Material characterization of ICCAs

**Fig. 2:**
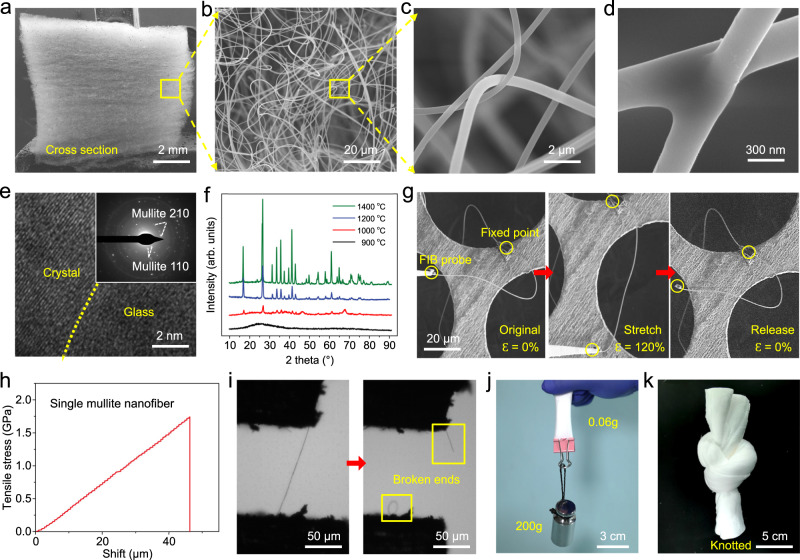
Material characterization of ICCAs. **a**, **b** SEM image of ICCAs at different magnifications in the cross section showing a knitted crimped-nanofibre structure with abundant entanglements. **c**, **d** SEM image of the interknitted structure and a crosslinking point between nanofibres. **e** Transmission electron microscopy (TEM) and high-angle annular dark-field STEM (HAADF-STEM) images of a mullite nanofibre. **f** XRD spectra of the mullite fibres. **g** Crimped nanofibre stretched from its original structure up to 120% strain without fracture. **h** Tensile stress-strain curves of a single ceramic nanofibre. **i** Images of the fibre before and after tensile fracture. **j**, **k** The ICCAs can be stretched and knotted without pulverization.

We first chose mullite as a sample for our proof-of-concept study in consideration of its thermal stability. The obtained ICCAs display distinctive structures, as shown in the scanning electron microscope (SEM) images (Fig. 2a–d). In a marked contrast to the lamellar structures of traditional ceramic fibrous materials in cross section and the pearl necklace-like structure of traditional ceramic aerogels, the ICCAs showed a 3D interwoven crimped-nanofibre structure based on the randomly entangled crimped-fibre framework including crimped fibres locked with each other and bonding points, which could be attributed to adhesion and fusion between two precursor fibres next to each other during calcination. Meanwhile, the average diameter of mullite nanofibres was 290 ± 30 nm (Supplementary Fig. 9).

We also characterized the chemical composition and crystallinity of a single ceramic fibre. From the relevant energy-dispersive X-ray spectroscopy (EDS) mapping results, Al, Si, and O elements were homogenously distributed in the ceramic nanofibres (Supplementary Fig. 10). The scanning transmission electron microscopy (STEM) and selected-area electron diffraction (SAED) pattern observations revealed that the obtained ceramic nanofibres were composed of numerous fine mullite grains with grain boundaries and glass phases (Fig. 2e). To better investigate the crystallization process of ICCAs, we performed thermogravimetric analysis (TGA), and the date revealed the thermal decomposition of the precursors with a weight loss of 50% and the formation of ceramic phases at approximately 1000 °C (Supplementary Fig. 11). The characteristic diffraction peaks of mullite in the X-ray diffraction (XRD) pattern further indicated the formation of mullite after calcination at 1000 °C. The crystalline structure was still a single mullite crystal even after heating at 1400 °C, indicating that the ICCAs exhibited thermal stability (Fig. 2f). Moreover, the pronounced Al *2p*, Si *2p*, and O *1* *s* peaks in the X-ray photoelectron spectroscopy (XPS) spectrum showed a single peak distribution, which confirmed the formation of pure mullite^39,40^ (Supplementary Fig. 12).

More interestingly, we found that the crimped mullite nanofibre is stretchable, as revealed by using focused ion beam-SEM (FIB-SEM). As shown in Fig. 2g and Supplementary Movie 3, the two bonding points between the crimped ceramic nanofibre and FIB probe could be stretched from their original distance to 120% tensile strain without fracture of the nanofibre. Figure 2h,–i shows the tensile stress-strain curves of the straight nanofibre. The tensile strength of the mullite nanofibre reached 1.74 GPa, rendering the ceramic nanofibres robust for preventing breakage while the aerogels were stretched. Therefore, based on the structure of ICCAs and the intrinsic performances of the single ceramic nanofibre, the ICCAs exhibited a series of superior features. (i) Ultralightweight: A piece of an 18-cm^3^ ICCA could be placed on the petal of a flower, and the minimum density of ICCAs was approximately 1.5 mg cm^−3^ corresponding to a porosity of 99.95% (Supplementary Fig. 13). (ii)Superior stretchability, flexibility and elasticity: The fabricated ICCAs exhibited stretchability and could be stretched and knotted without any visible breakage/fracture (Fig. 2j–k). In particular, ICCAs (density of 6 mg cm^−3^) with a weight of 0.06 g could withstand up to ~3300 times their own weight without fracture.

### Temperature-invariant stretchability, flexibility and compressibility

**Fig. 3:**
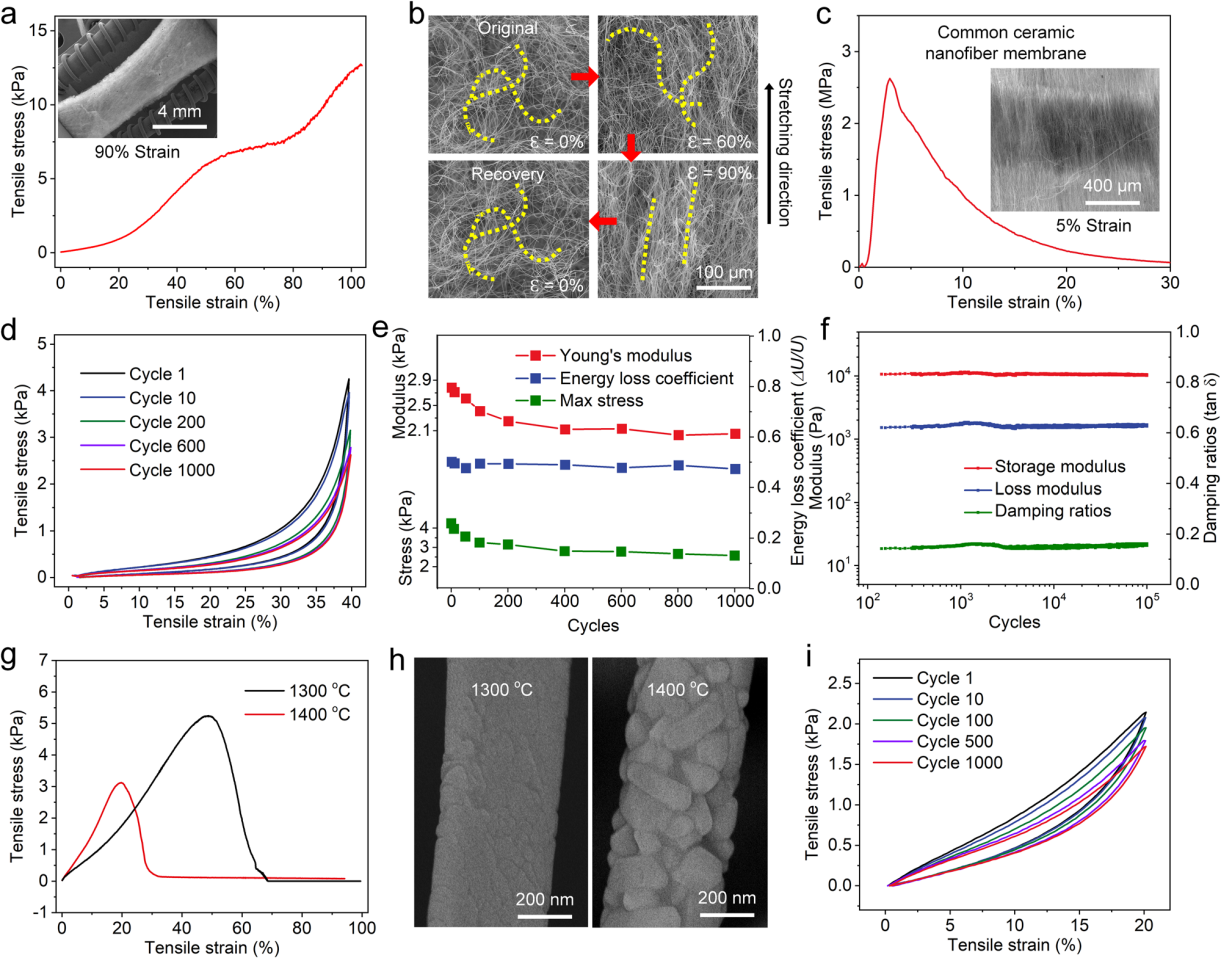
Temperature-invariant stretchability of the ICCAs. **a** Tensile stress–strain curves of ICCAs. (insert) SEM image of ICCAs with 90% tensile strain. **b** In situ stretching of ICCAs, showing the stretching-recovery performance. **c** Tensile stress–strain curves of a common ceramic nanofibre membrane. (insert) SEM image of the membrane with 5% tensile strain. **d** One-thousand-cycle tensile test with 40% tensile strain. **e** Young’s modulus, energy loss coefficient, and maximum stress versus tensile cycle. **f** Storage modulus, loss modulus, and damping ratio of ICCAs during 100,000 tensile-release fatigue cycles; the oscillatory strain was 5%. **g** Tensile stress–strain curves of ICCAs after holding them at high-temperature conditions for 1 h. **h** SEM images of ICCAs after treatment at 1300 °C and 1400 °C for 1 h. **i** One-thousand-cycle tensile test with 20% tensile strain after holding at 1300 °C for 1 h.

We further evaluated the superior mechanical behavior of ICCAs over a wide range of temperatures by carrying out a series of tests on a dynamic mechanical analyser (DMA) instrument. Figure 3a shows that the ICCAs could be stretched from their original morphology to 100% tensile strain without fracture. The tensile fracture stress was 12.7 kPa. According to the tensile stress-strain curve, we observed three characteristic stages in the loading-unloading process: a relatively linear elastic regime when ε < 50%, a subsequent nearly flat stress plateau when 50% < ε < 80%, and a final regime when ε > 80% in which σ increased sharply. To gain deeper insight into the peculiar stretchability, we examined the microstructural evolution by performing in situ SEM tensile test. The ICCAs without tensile strain exhibited a 3D interwoven crimped-nanofibre structure. When the tensile strain reached 90%, declinations, deformations, interlockings and orientations caused by straightening appeared in the crimped nanofibres of the ICCAs, preventing structural collapse (Fig. 3b and Supplementary Fig. 14). In contrast, the ceramic membrane with lamellar structure and straight nanofibres had smaller final strain (5%) and large module (61 MPa)(Fig. 3c), and it experienced a fracture that appeared to be caused by slippage of the straight nanofibres (Fig. 3c insert), confirming the importance of the 3D interwoven crimped-nanofibre structure. Upon further investigation, we considered that the mechanical properties of ceramic nanofirous aerogels are dictated by the microstructure of aerogel and the intrinsic properties of ceramic nanofibres. First, the ceramic nanofibres are long, crimped, flexible and robust, preventing the ceramic nanofibre from breaking before the aerogel fractures. Second, the modules (2.7 kPa) of ICCAs were substantially lower than the value of ceramic nanofirous membrane indicated that the low density and high porosity structure of the ICCAs provide sufficient space for ceramic nanofibres to deflect rather than stress concentration when the aerogel suffers large strain. Third, the entanglement structure is conducive to maintaining the network structure, and the tension would be distributed from one nanofibre to many others at nodal points of the entanglement structure when the ceramic aerogel was stretched (Supplementary Fig. 15).

Subsequently, we demonstrated that the ICCAs could withstand 1000 tensile-release cycles at a strain of 40% (Fig. 3d). The aerogels displayed invisible plastic deformation, and the maximum stress remained at 60% of that in the first cycle. The constant energy loss coefficient (~ 0.47) during cyclic tensile testing indicated the structural robustness (Fig. 3e). More interestingly, the ICCAs exhibited amazing tensile-recovery fatigue resistance behaviour. As presented in Fig. 3f, after being stretched for 100 000 cycles at an oscillatory strain of 5%, storage modulus, loss modulus and damping ratio (~ 0.16) remained almost identical, highlighting stretching elasticity and fatigue resistance. Meanwhile, the ICCAs performed constant viscoelastic properties as same as the sample without treatment over a wide angular frequency of 0.1 to 100 rad s^−1^ (Supplementary Fig. 16), indicating a high dynamic stretching elastic response. Furthermore, a buckling-recovery test with increasing strain to 90% and a 1,000-cycle buckling-recovery test were performed to demonstrate the flexibility of the ICCAs (Supplementary Fig. 17). The aerogels did not obviously fracture even when the buckling ratio was up to 95%, or after buckling deformation for 1,000 cycles at a buckling strain of 90%, indicating a high flexibility. Moreover, the ICCAs exhibited large compression recovery and robust compressive fatigue tolerance up to 1000 cycles at a compressive strain of 60% (Supplementary Fig. 18).

ICCAs that combine the peculiar properties of the interwoven crimped-nanofibre structure with a mullite nature are anticipated to provide temperature-invariant stretching elasticity. As shown in Supplementary Fig. 19, the dynamic storage tensile modulus, the dynamic loss tensile modulus and the damping ratio remained nearly invariable from -100 to 500 °C. For further investigation of the mechanical stability at extreme temperatures, we heated the ICCAs at temperatures of 1300 °C and 1400 °C for 1 h. After calcination at 1300 °C, the ICCAs showed superb ductility with a tensile strain up to 48.3%. The aerogels calcinated at 1400 °C had a smaller final strain (23.2%) (Fig. 3g), which might be attributed to the increase in defects in nanofibres produced by excessive crystallite growth during high-temperature treatment (Fig. 3h and Supplementary Fig. 20). Upon further investigation, we found that the crystallinity of ICCAs increased gradually with the increase of treatment temperature. Meanwhile, the final strain decreased and modulus of ICCAs increased with the increase of crystallinity indicating that modulus of ceramic nanofibre increased and brittle fracture would occur more easily after ceramic nanofibre buckling (Supplementary Fig. 21). The results revealed that the aerogels can preserve superior stretchability after treatment at deep cryogenic or ultrahigh temperatures. Subsequently, we explored the stretching elasticity of the sample annealed at 1300 °C for 1 h. More importantly, this aerogel can also sustain up to 1,000 tensile-release fatigue cycles with a 20% tensile strain (Fig. 3i). Moreover, the aerogels can be completely restored to their original shape when bent or compressed while being either exposed to the flame of a butane blowtorch or immersed in liquid nitrogen (Supplementary Fig. 22–23). Thus, the temperature-invariant stretchability, flexibility and compressibility of ICCAs from -196 to 1400 °C have been demonstrated.

### Thermal insulation and fire resistance

**Fig. 4:**
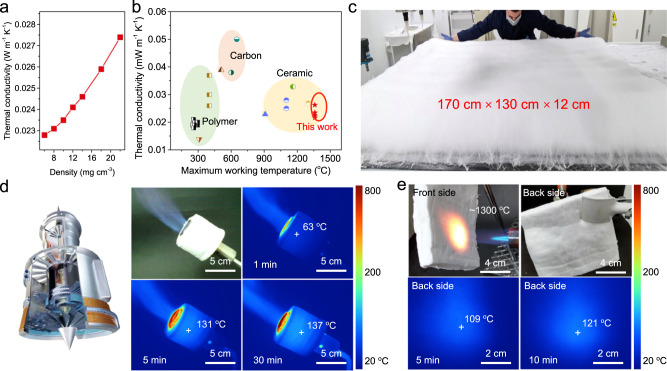
Thermal insulation properties of ICCAs. **a** Thermal conductivity of the ICCAs as a function of density. **b** Comparison of the thermal conductivity and maximum working temperature among different aerogel-like materials. **c** Optical image of a large-size precursor ICCA. **d** Infrared images of a butane nozzle protected by ICCAs during a 30 min heating process, the colored scale bar shows the temperature. **e** Optical and infrared images of ICCAs exposed to a butane blowtorch for 10 min.

In our previous studies, we found that the total thermal conductivity (λ_t_) is mostly affected by the solid thermal conductivity (λ_s_) and the gaseous thermal conductivity (λ_g_), which depends on the porosity of materials at ambient pressure and room temperature^14,41^. Generally, porous materials can exhibit remarkably increased thermal resistance through the combination of restricted convection and decreased conduction at room temperature provided by the nanofibrous structure and ultralight characteristics^42^. As a result, our ultralight nanofibrous aerogels exhibited thermal insulating performance. Figure 4a shows a plot of λ_t_ as a function of the density of ICCAs. λ_t_ was as low as 0.0228 W m^−1^ K^−1^ with a density of 6 mg cm^−3^ under ambient conditions, which could be attributed to the ultralow diameter of the ceramic nanofibre and high porosity (99.8%). This value is lower than the thermal conductivity of air. When the density was increased to 22 mg cm^−3^, λ_t_ was increased slightly to 0.0274 W m^−1^ K^−1^ by virtue of the almost constant porosity (99.27%). This low thermal conductivity in combination with the high degree of temperature-invariant stretchability and flexibility allows ICCAs to be applied as thermal insulation materials under extreme environments beyond the reach of conventional porous insulation materials (Fig. 4b and Supplementary Table 2). (i) Most porous polymeric insulation materials, such as polybenzazole aerogels and polysilsesquioxane aerogels usually exhibit a relatively low thermal conductivity, but cannot withstand temperatures above 500 °C (green zone)^40,43,44^. (ii) Carbon materials show a relatively high heat resistance, but still cannot withstand a high-temperature oxidizing atmosphere (orange zone)^45–47^. (iii) Compared with previously reported advanced ceramic insulators, the ICCAs exhibited a λ value among the lowest values reported with robust structural stability up to 1400 °C, indicating that they are more attractive for extreme thermal insulation^1,15,17,21^. Moreover, the macroscopic product can be easily and quickly prepared by pilot equipment. As shown in Fig. 4c and Supplementary Fig. 24, a ceramic nanofibrous aerogel precursor 170 cm long, 130 cm wide, and 12 cm high weighing 313 g were produced in one hour.

The thermal insulation materials used to protect equipment and fuel tanks in aero-engines or scramjet engines should withstand high temperatures or even direct contact with a flame^48^. Therefore, we used infrared camera observations to monitor the dynamic temperature distribution of ICCAs upon heating and exposure to flames to further evaluate the practical thermal insulation performance under extreme conditions. First, we wrapped ICCAs that had the thickness of 10 mm and a density of 8 mg/cm^3^ around the nozzle of a butane blowlamp(Fig. 4d). When the butane blowlamp was turned on, we took thermal-infrared images in real time to characterize the time-dependent high-temperature thermal insulation property. After heating for 5 min, the temperature of the nozzle rapidly exceeded the limitation of the equipment (800 °C), while the temperature of the ICCAs exhibited a slow rise to a maximum temperature of approximately 137 °C. Remarkably, the temperature outside of the ICCAs remained nearly constant even after 30 min, indicating that the aerogels are superior thermal insulators. Subsequently, a piece of ICCA with a density of 22 mg/cm^3^ and a thickness of 20 mm was directly heated by a butane blowlamp flame (approximately 1300 °C^37^). Similarly, the backside temperature slowly increased to 121 °C after 10 min, demonstrating the robust fire resistance of the ICCAs (Fig. 4e). These results indicated that our aerogels present a combination of thermal insulation and robust structural stability and stretchability that offers extensive potential applicability in the thermal insulation field under extreme conditions, such as fire suits for emergency rescue and in aviation and aerospace industries.

## Discussion

The successful synthesis of ICCAs using 3D reaction electrospinning provides a methodology to explore the formation of 3D interwoven crimped-nanofibre structured ceramic aerogel materials. Here, mullite-based nanofibrous aerogels served as model systems for a proof of concept. The rate of gelation of the sol jet was controlled to achieve precise control of the jet shape. The final ICCAs exhibit superior performance such as ultralightweight, flexibility, stretchability, compressibility, fatigue tolerance, and ultralow thermal conductivity over a very wide temperature range. Considering the ease of optimization of sol solutions based on the sol-gel method and the diversity of the nanofibres, our work will provide a versatile platform for designing new types of nanofibrous aerogels for various applications. Moreover, we showed that the ICCAs can be easily scaled up in pilot equipment. Due to their integrated properties, we can expect that the ICCAs will have broad technological and engineering implications for personal protective equipment, thermal protection systems in space vehicles and flexible wearable electronics.

## Methods

### Fabrication of ICCAs

Stretchable and flexible mullite nanofibre aerogels were prepared through sol-gel and electrospinning. First, the mullite sol with a molar composition of aluminum chloride hexahydrate (AlCl_3_·6H_2_O, 97%, Aladdin): aluminum isopropoixide (AIP, Al(C_3_H_7_O)_3_, 98.5%, Aladdin): tetraethyl orthosilicate (TEOS, Si(OC_2_H_5_)_4_, 98%, Greagent): oxalic acid (99%, Aladdin): H_2_O: ethanol (99.7%, Greagent) = 1 :2.5 :1.16 :0.033 :60 :16 was prepared through hydrolysis and condensation, which was stirred at 25 °C for 10 h. Subsequently, a series of sols were obtained by decreasing the pH of the unprotonated sol from 2.6 to 1.7 and 0.8 through dropwise addition of ethanol-diluted hydrochloric acid solution. PEO (Mn = 60 w, Aladdin) was added to the mullite sol with a sol/PEO weight ratio of 1000: 1 with stirring for another 8 h. Then, the mullite nanofibre aerogel precursors were directly produced by using the obtained solution with the electrospinning method.

During electrospinning, the spinning solution was injected at a speed of 10 ml/h and stretched by an applied voltage of 25 kV. The mullite nanofibres were collected on the surface of the metallic rotating covered by aluminium foil at a 220-mm receiving distance. Furthermore, the humidity (40 ± 2%) and relevant temperature (22 ± 2 °C) during electrospinning were controlled. Moreover, the spun specimen was immediately calcined in a muffle furnace. The process of calcination can be divided into four stages: rapid heating at 15 °C min^−1^ from 20 to 1000 °C; maintenance at 1000 °C for 1 h; heating at 10 °C min^−1^ from 1000 °C to the set temperature (1100~1500 °C); and maintenance at the set temperature for 1 h. Other mullite nanofibre aerogels with different densities were manufactured via adjusting the addition of water and ethanol. Except as noted, mullite nanofibre aerogels with the density of 6 mg cm^−3^ were used to perform the structural and physical property tests.

### Characterization

The microstructure of ICCAs was characterized by FE-SEM (S-4800) and TEM (JEM-2100). Optical images of ICCAs and sol solution were recorded by a digital video camera (Canon M50). An infrared thermal camera (Fluke TiX560) was used to take the infrared images of ICCAs. The chemical structure of ICCAs were tested via XPS (PHI 5000 C ESCA), TGA(TA SDT Q600 TG-DSC analyzer) and XRD (Bruker D8 ADVANCE). The single fibre tensile test involves the following parts: (a) preparing the dispersion of ICCAs to obtain single nanofibre; (b) a thin layer of ceramic binder is applied to the fibre placement area of the fixed sample table. Then the fibre samples are received and left to cure for 12 h; (c) put the sample stage gently into the fixture and start the test. The size of particles was characterized by DLS (Malvern zetasizer nano ZEN3700). The thermal conductivity of ICCAs was characterized by a Hot Disk instrument (TPS2500S, Switzerland). All the mechanical tests were performed using a DMA instrument (TA-Q850). The temperature and the frequency-dependent tensile properties of ICCAs were assessed with an oscillatory strain of 3%.

Molecular computational details: We used the Gaussian 16 ver. C.01 and Multiwfn ver. 3.7 were to study all DFT calculations. By using Visual Molecular Dynamics (VMD) ver. 1.9.3. Geometries optimization of ground state, we obtained the corresponding visualization picture. Through the unrestricted DFT-B3LYP/6-311 + G(d, p) level of theory, we performed geometric optimization in polarizable continuum model in H_2_O solvent. Based on the optimized structure above, we calculated the excitations at the DFT-B3LYP/6-311 + G(d, p) level of theory in H_2_O solvent model and obtained the hole/electron heat map by Multiwfn 3.6 programs. At unrestricted DFT-B3LYP/6-311 + G(d, p) level of theory in a H_2_O solvent, single-point energy was performed on the CHELPG5 ESP population analysis.

## Supplementary information

Supplementary Information

Description of Additional Supplementary Files

Supplementary Movie 1

Supplementary Movie 2

Supplementary Movie 3

## Data Availability

The data generated in this study are provided in the Manuscript and Supplementary Information. All other data that support the findings of this study are available from the corresponding author upon a request.

## References

[1] Xu X (2019). Double-negative-index ceramic aerogels for thermal superinsulation. Science.

[2] Zhao SY (2020). Additive manufacturing of silica aerogels. Nature.

[3] Ziegler C (2017). Modern Inorganic Aerogels. Angew. Chem. -Int. Ed..

[4] He YL, Xie T (2015). Advances of thermal conductivity models of nanoscale silica aerogel insulation material. Appl. Therm. Eng..

[5] An L (2020). An All-Ceramic, Anisotropic, and Flexible Aerogel Insulation Material. Nano Lett..

[6] Huber L, Zhao SY, Malfait WJ, Vares S, Koebel MM (2017). Fast and minimal-solvent production of superinsulating silica aerogel granulate. Angew. Chem. -Int. Ed..

[7] Baumann TF (2005). Synthesis of high-surface-area alumina aerogels without the use of alkoxide precursors. Chem. Mat..

[8] Madyan OA, Fan M, Feo L, Hui D (2016). Enhancing mechanical properties of clay aerogel composites: An overview. Compos. Pt. B-Eng..

[9] Guo F (2018). Highly stretchable carbon aerogels. Nat. Commun..

[10] Yeo SJ, Oh MJ, Yoo PJ (2019). Structurally controlled cellular architectures for high-performance ultra-lightweight materials. Adv. Mater..

[11] Meza LR, Das S, Greer JR (2014). Strong, lightweight, and recoverable three-dimensional ceramic nanolattices. Science.

[12] Zhang M (2020). Conductive and Elastic TiO_2_ Nanofibrous Aerogels: A new concept toward self-supported electrocatalysts with superior activity and durability. Angew. Chem. -Int. Ed..

[13] Wan WC, Zhang RY, Ma MZ, Zhou Y (2018). Monolithic aerogel photocatalysts: a review. J. Mater. Chem. A.

[14] Dou L (2020). Interweaved cellular structured ceramic nanofibrous aerogels with superior bendability and compressibility. Adv. Funct. Mater..

[15] Si Y, Wang XQ, Dou LY, Yu JY, Ding B (2018). Ultralight and fire-resistant ceramic nanofibrous aerogels with temperature-invariant superelasticity. Sci. Adv..

[16] Wang F (2020). In situ synthesis of biomimetic silica nanofibrous aerogels with temperature-invariant superelasticity over one million compressions. Angew. Chem. -Int. Ed..

[17] Zhang XX (2020). Ultrastrong, superelastic, and lamellar multiarch structured zro2-al2o3 nanofibrous aerogels with high-temperature resistance over 1300 °C. ACS Nano.

[18] Liu RL (2019). Ultralight, thermal insulating, and high-temperature-resistant mullite-based nanofibrous aerogels. Chem. Eng. J..

[19] Zhang ES (2021). Insulating and robust ceramic nanorod aerogels with high-temperature resistance over 1400 °C. ACS Appl. Mater. Interfaces.

[20] Su L (2020). Anisotropic and hierarchical SiC@SiO_2_ nanowire aerogel with exceptional stiffness and stability for thermal superinsulation. Sci. Adv..

[21] Jia C (2020). Highly compressible and anisotropic lamellar ceramic sponges with superior thermal insulation and acoustic absorption performances. Nat. Commun..

[22] Wang Y (2020). Self-assembly of ultralight and compressible inorganic sponges with hierarchical porosity by electrospinning. Ceram. Int..

[23] Kim J, Zhang G, Shi M, Suo Z (2021). Fracture, fatigue, and friction of polymers in which entanglements greatly outnumber cross-links. Sci. (N. Y., N. Y.).

[24] Mackanic DG, Chang TH, Huang ZJ, Cui Y, Bao ZN (2020). Stretchable electrochemical energy storage devices. Chem. Soc. Rev..

[25] Schutt F (2017). Hierarchical self-entangled carbon nanotube tube networks. Nat. Commun..

[26] Feng X (2011). Stretchable ferroelectric nanoribbons with wavy configurations on elastomeric substrates. ACS Nano.

[27] Eckel ZC (2016). 3D PRINTING Additive manufacturing of polymer-derived ceramics. Science.

[28] Moon S (2021). 3D jet writing of mechanically actuated tandem scaffolds. Sci. Adv..

[29] Jordahl JH (2018). 3D Jet Writing: Functional microtissues based on tessellated scaffold architectures. Adv. Mater..

[30] Wang J (2022). Polymer templates effects on microstructure and mechanical properties of electrospun mullite nanofibers. Ceram. Int..

[31] Zhang SC (2019). Direct electronetting of high-performance membranes based on self-assembled 2D nanoarchitectured networks. Nat. Commun..

[32] Zhang SC, Liu H, Yu JY, Li BY, Ding B (2020). Multi-functional flexible 2D carbon nanostructured networks. Nat. Commun..

[33] Shan HR (2017). Hierarchical porous structured SiO_2_/SnO_2_ nanofibrous membrane with superb flexibility for molecular filtration. ACS Appl. Mater. Interfaces.

[34] Liu LC, Moreno L, Neretnieks I (2009). A dynamic force balance model for colloidal expansion and its DLVO-based application. Langmuir.

[35] Oguzlu H, Danumah C, Boluk Y (2017). Colloidal behavior of aqueous cellulose nanocrystal suspensions. Curr. Opin. Colloid Interface Sci..

[36] Wyss HM, Innerlohinger J, Meier LP, Gauckler LJ, Glatter O (2004). Small-angle static light scattering of concentrated silica suspensions during in situ destabilization. J. Colloid Inter. Sci..

[37] Dong X (2017). Electrospun mullite nanofibres derived from diphasic mullite sol. J. Am. Ceram. Soc..

[38] Wang HL (2017). Ultralight, scalable, and high-temperature-resilient ceramic nanofibre sponges. Sci. Adv..

[39] Chen ZX (2015). Electrospun mullite fibres from the sol-gel precursor. J. Sol.-Gel Sci. Technol..

[40] Schneider H, Schreuer J, Hildmann B (2008). Structure and properties of mullite - A review. J. Eur. Ceram. Soc..

[41] Wicklein B (2015). Thermally insulating and fire-retardant lightweight anisotropic foams based on nanocellulose and graphene oxide. Nat. Nanotechnol..

[42] Hu F, Wu SY, Sun YG (2019). Hollow-structured materials for thermal insulation. Adv. Mater..

[43] Qian ZC (2018). Fire-resistant, ultralight, superelastic and thermally insulated polybenzazole aerogels. J. Mater. Chem. A.

[44] Zou FX (2016). Robust and superhydrophobic thiourethane bridged polysilsesquioxane aerogels as potential thermal insulation materials. J. Mater. Chem. A.

[45] Zhang XH, Li W, Song PY, You B, Sun G (2020). Double-cross-linking strategy for preparing flexible, robust, and multifunctional polyimide aerogel. Chem. Eng. J..

[46] Liang CY, Wang ZJ (2019). Eggplant-derived SiC aerogels with high-performance electromagnetic wave absorption and thermal insulation properties. Chem. Eng. J..

[47] Zhang QQ (2017). Flyweight, Superelastic, Electrically conductive, and flame-retardant 3D multi-nanolayer graphene/ceramic metamaterial. Adv. Mater..

[48] Dong YW, Wang ET, You YC, Yin CP, Wu ZP (2019). Thermal protection system and thermal management for combined-cycle engine: review and prospects. Energies.

