# Supplementary material for: Direct synthesis of highly stretchable ceramic nanofibrous aerogels via 3D reaction electrospinning
Source: Nat Commun. 2022 May 12;13:2637. doi: 10.1038/s41467-022-30435-z (PMC9098874; doi:10.1038/s41467-022-30435-z)
Supplement: Supplementary file 1 — Supplementary Information [file 41467_2022_30435_MOESM1_ESM.pdf]

Supplementary information

Direct synthesis of highly stretchable ceramic nanofibrous aerogels via 3D reaction electrospinning

Xiaota Cheng, Yi-tao Liu, Yang Si*, Jianyong Yu, Bin Ding*

Xiaota Cheng, Yi-tao Liu, Yang Si, Jianyong Yu, Bin Ding
Innovation Center for Textile Science and Technology, College of Textiles, Donghua University, Shanghai 201620, China

Email: yangsi@dhu.edu.cn; binding@dhu.edu.cn

Supplementary Figures

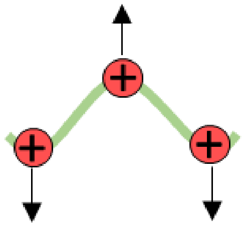

Supplementary Fig. 1 Illustration of whipping instability. Diagram showed that high surface potential results in deflection of the jet from the centerline, which is premise of whipping instability.

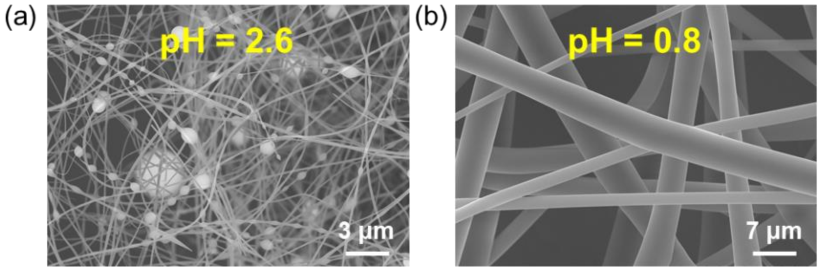

Supplementary Fig. 2 Microstructural contrast of fibres prepared with different pH. SEM images of ceramic fibres fabricated with different values of pH. (a) pH = 2.6 and (b) pH = 0.8.

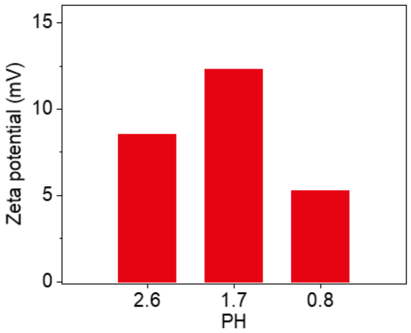

Supplementary Fig. 3 Characterization of stability of spinning solutions. Zeta potential of three spinning solutions with different values of pH.

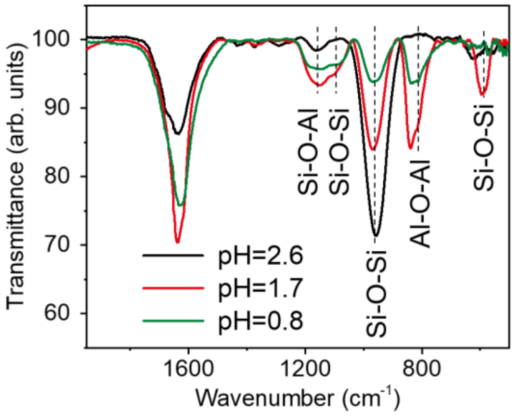

Supplementary Fig. 4 Comparison of degree of condensation via FTIR spectra. FTIR spectra of three precursor fibres fabricated with different values of pH. Precursor fibres: as-spun fibres without any treatment.

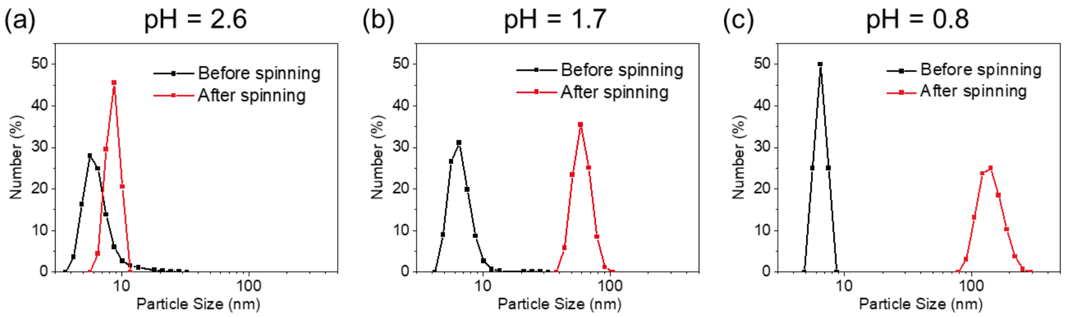

Supplementary Fig. 5 Comparison of degree of condensation via DLS test. Sizes of colloidal particles formed by dissolving three precursor fibres prepared with different values of pH (a) 2.6, (b) 1.7 amd (c) 0.8 in water. Precursor fibres: as-spun fibres without any treatment.

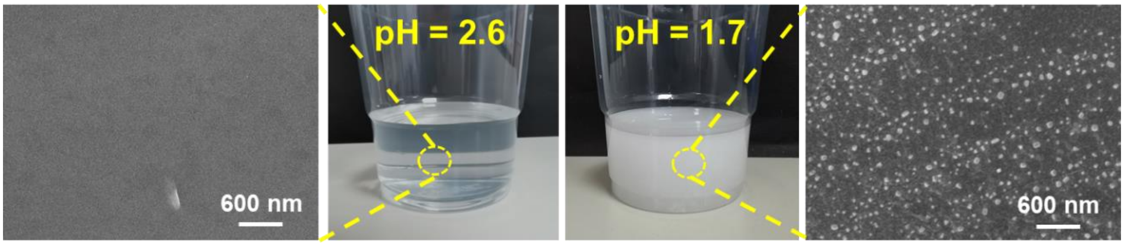

Supplementary Fig. 6 Comparison of degree of condensation via images. Optical and SEM images of solutions formed by dissolving precursor fibres prepared with pH 2.6 (left) and 1.7 (right) in water.

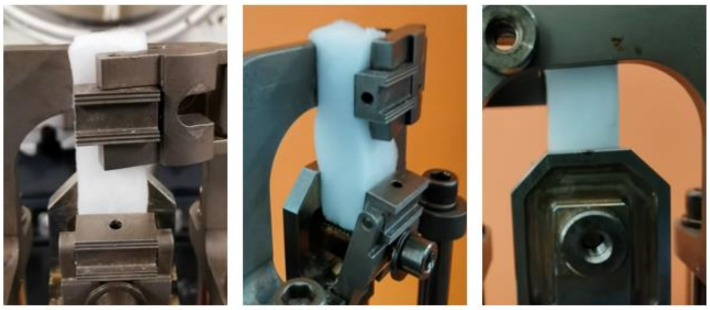

Supplementary Fig. 7 Original state of tensile test. Optical image of the fibrous aerogel before being stretched in DMA.

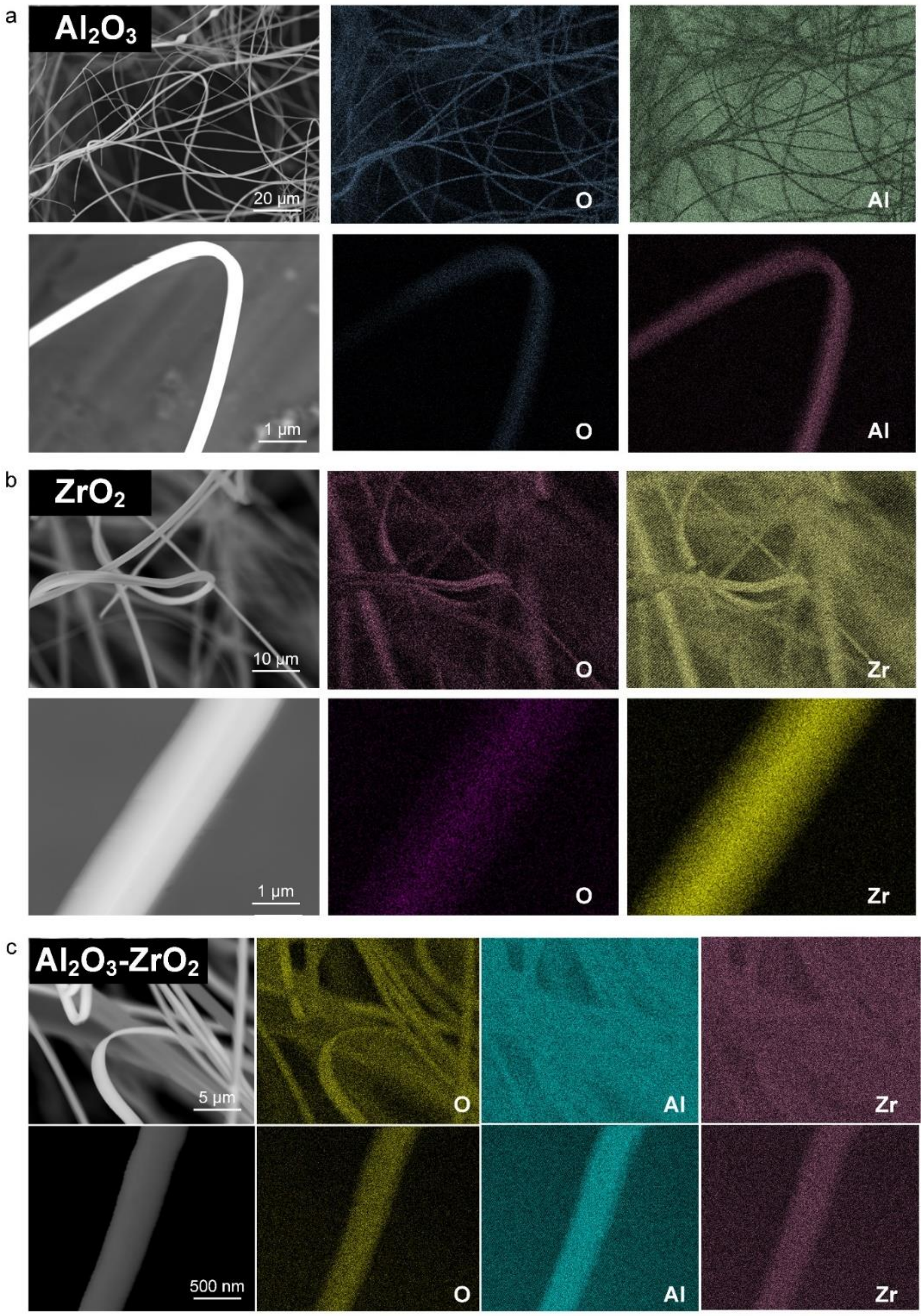

Supplementary Fig. 8 Characterization of different ceramic aerogels. SEM images and EDS mappings results of (a) Al_2O_3, (b)ZrO_2 and (c) Al_2O_3-ZrO_2 ceramic nanofibrous aerogels.

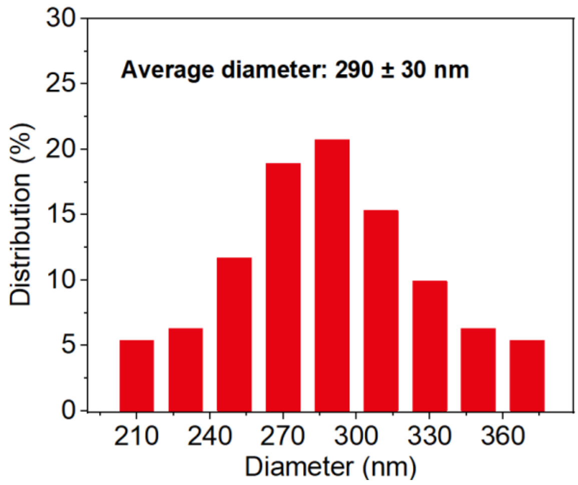

Supplementary Fig. 9 Diameter distribution. The diameter distribution of mullite nanofibre prepared with pH 1.7.

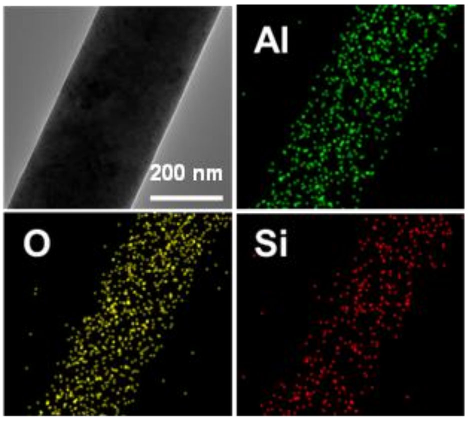

Supplementary Fig. 10 Characterization of single nanofibre. TEM and EDS mapping images of single mullite nanofibre.

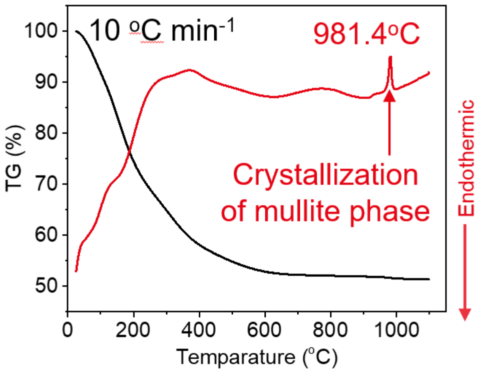

Supplementary Fig. 11 Analysis of calcination process. TGA and DTA traces of the aerogel precursors.

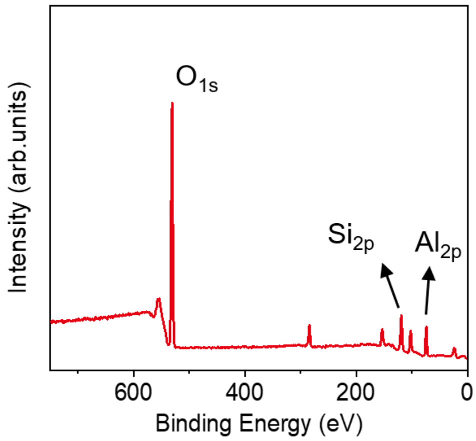

Supplementary Fig. 12 Component analysis. XPS spectra of the mullite fibres.

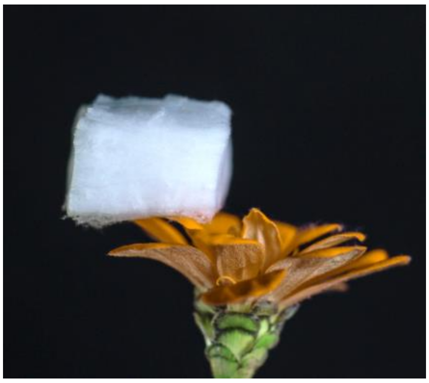

Supplementary Fig. 13 Display of ultralight property. Optical image showing a 20-cm^3 ICCA (ρ =1.5 mg cm^{-3}) standing on a flower.

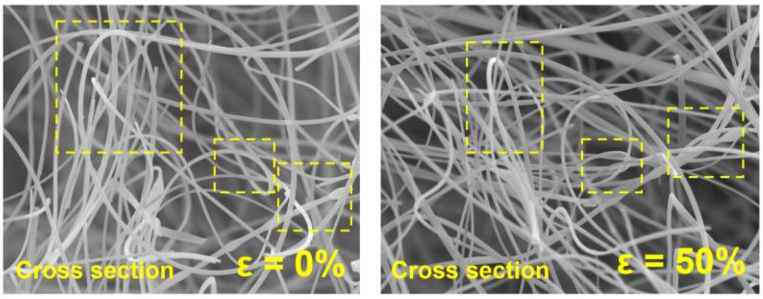

Supplementary Fig. 14 In situ tensile test. SEM images of ICCAs with (left) original state and (right) 50% tensile strain.

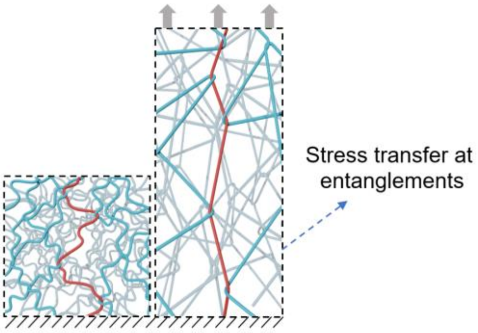

Supplementary Fig. 15 Illustration of tensile process. Diagram showed that stress transfer at entanglements to other nanofibres.

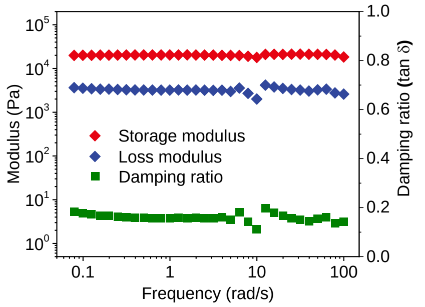

Supplementary Fig. 16 Viscoelastic test of ICCAs. The frequency dependence of the storage modulus, loss modulus, and damping ratio for ICCAs (tensile oscillatory strain of 3%).

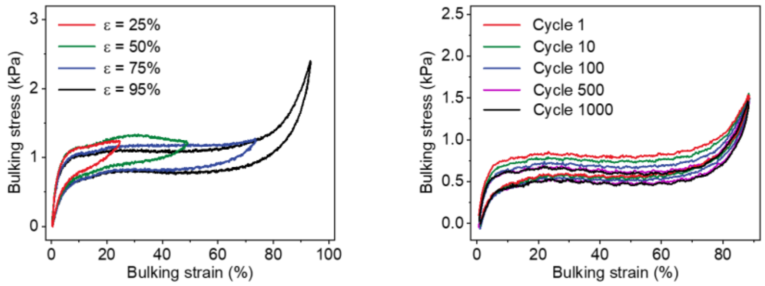

Supplementary Fig. 17 Bulking test of ICCAs. Stress-strain curves of at 25, 50, 75, and 95% bulking strains (left) and 1,000-cyclic fatigue test with 90% bulking strain (right).

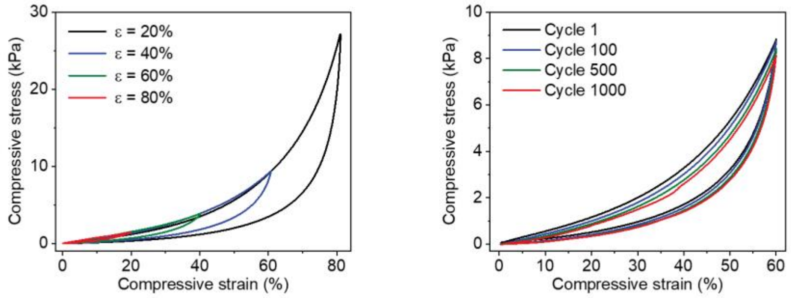

Supplementary Fig. 18 Compression test of ICCAs. Stress-strain curves of at 20, 40, 60, and 80% compressive strains and 1,000-cyclic fatigue test with 60% compressive strain.

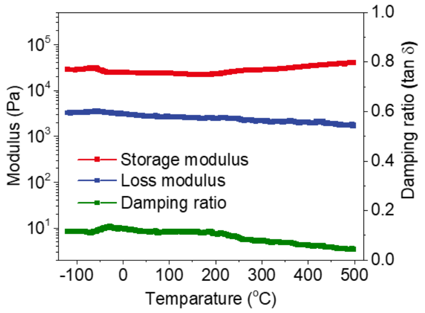

Supplementary Fig. 19 Temperature-invariant stretchability. The temperature dependence of the storage modulus, loss modulus, and damping ratio for ICCAs, the oscillatory strain was 3%.

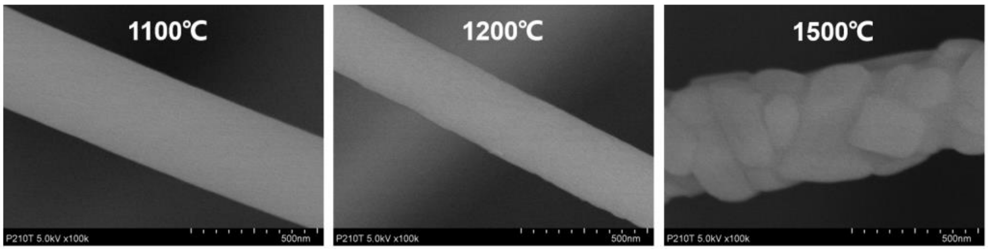

Supplementary Fig. 20 Characterization of mullite nanofibres treated at different temperatures. The SEM images of single fibre of ICCAs after treatment at 1100°C, 1200°C and 1500 °C for 1h.

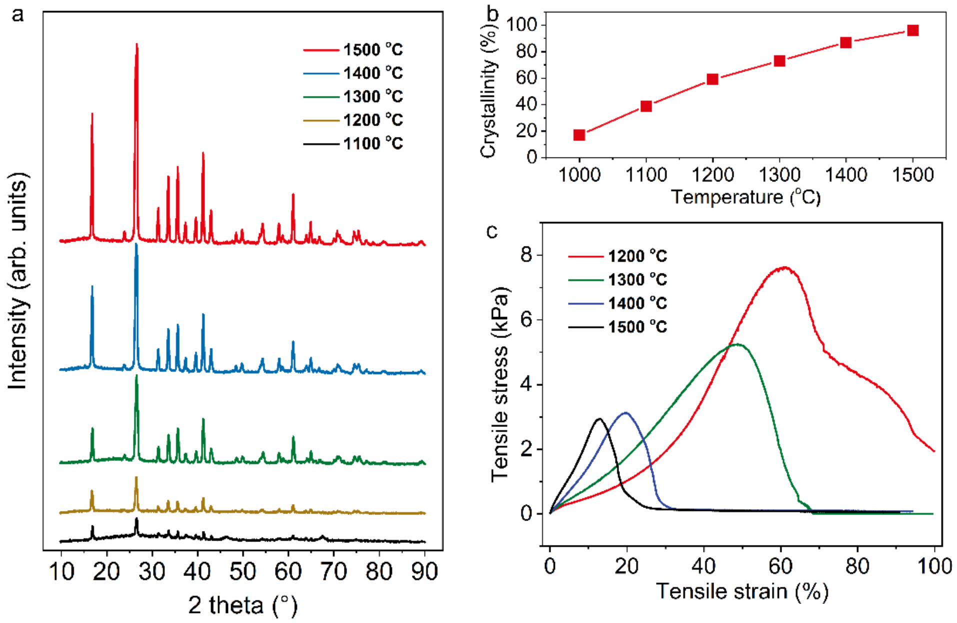

Supplementary Fig. 21 Correlation analysis between crystallinity and mechanics. (a) XRD patterns, (b) Crystallinity and (c) Tensile stress–strain curves of ICCAs calcinated at different temperature for 1 h.

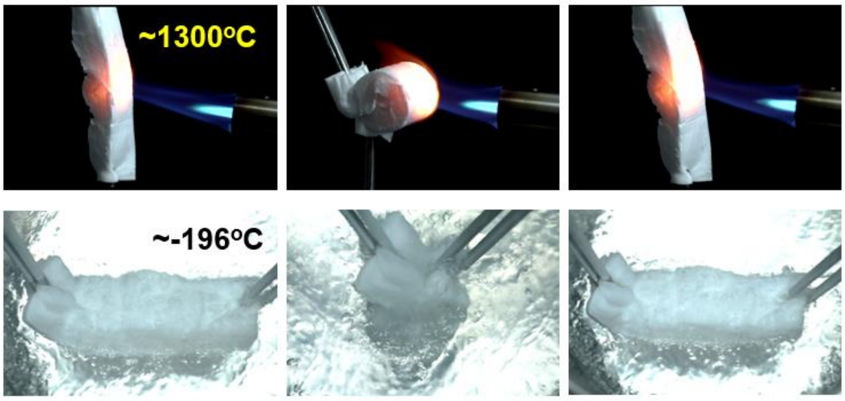

Supplementary Fig. 22 Temperature-invariant flexibility. Bending and recovery processes in butane blowtorch flame and liquid nitrogen.

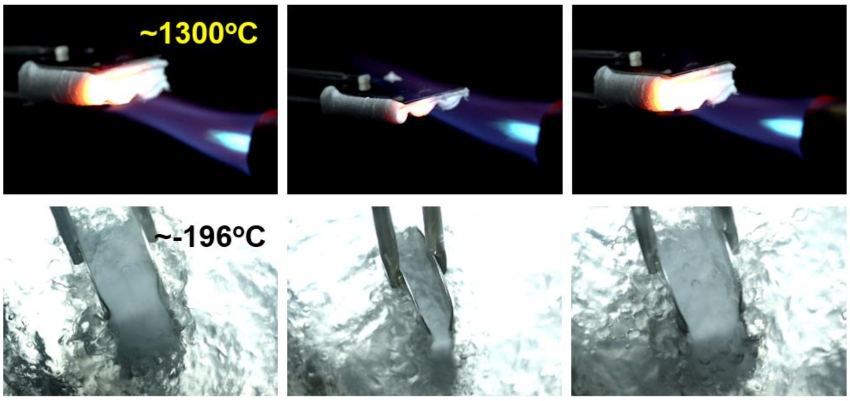

Supplementary Fig. 23 Temperature-invariant compressibility. Compression and recovery processes in butane blowtorch flame and liquid nitrogen.

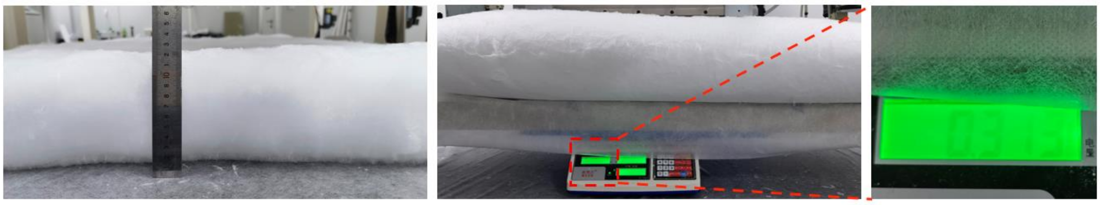

Supplementary Fig. 24 Large scale ICCAs. Thickness and weight of large size precursor ICCAs prepared by pilot equipment.

Supplementary Tables

Supplementary Table 1 Electronegativity of Si and distance between Si and O

	Protonated group	Unprotonated group	After condensation
Electronegativity of Si	6.756	-18.196	-11.746
Distance between Si and O	3.47112Å	1.64585Å	1.6578Å

Supplementary Table 2 The relevant properties of ICCAs and conventional porous insulation materials.

Materials	Maximum working temperature in air (^oC)	Thermal conductivity ($W\ m^{-1}\ K^{-1}$)
ICCAs	1400	0.022-0.027
Polysilsesquioxane aerogels	250-300	0.018-0.021
Nanocellulose and graphene oxide foams	350	0.015

Polybenzazole aerogels	400	0.026-0.037
Polyimide/rGO aerogel	550	0.04
SiC/C aerogel	600	0.038
Graphene/ceramic metamaterial	650	0.05
BN aerogel	900	0.024
SiO_2 nanofibre aerogel	1100	0.025-0.028
SiO_2-Al_2O_3 fibre sponges	1100	0.025
ZrO_2 fibre sponges	1300	0.027

Supplementary Notes

Determination of density of the aerogel: The density of the aerogel mentioned in this manuscript was measured on the basis of ISO 845:2006 standard: Cellular plastic and rubbers—Determination of apparent density. This standard is the most widely used for measuring the density of cellular materials. The density of the aerogel was calculated according to the equation (1):

$$Apparent\ density = \frac{Mass\ of\ solid\ constituents}{Volume\ of\ test\ sample} \quad (1)$$

The porosity of the aerogel was calculated based on the equation (2):

$$\eta = \frac{V_0 - m/\rho}{V_0} \times 100\% \quad (2)$$

where V_0 (cm^3) was the volume of the aerogel, m (g) and ρ (g cm^{-3}) were mass and density of solid constituent, respectively. The solid constituent of the aerogel was mullite, whose density was about 3.16 g cm^{-3}.

The testing process for tensile fracture of single ceramic nanofibre: First, we controlled the slit on the sample table with 50 μm distance, and applied glue to the edges of the slit. The sample of ICCAs was broken for obtaining an individual ceramic nanofibre, which was placed at the slit (as shown in Supplementary Fig. 25).

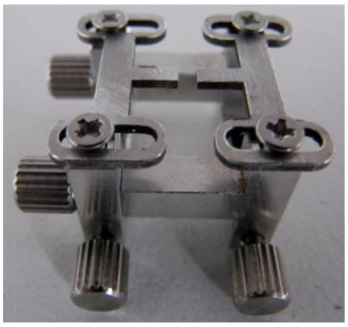

Supplementary Fig. 25 The picture of the sample table

Second, we calibrated the sensor in the sample storehouse (as shown in the Supplementary Fig. 25).

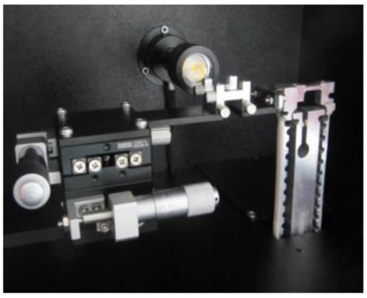

Supplementary Fig. 26 The picture of the sample storehouse.

Third, we loaded the sample table into the sample storehouse. Before testing, we cleared the force value in the software interface, gently put the sample table on the fixture and started the test.
