# Supplementary material for: Direct synthesis of highly stretchable ceramic nanofibrous aerogels via 3D reaction electrospinning
Source: Nat Commun. 2022 May 12;13:2637. doi: 10.1038/s41467-022-30435-z (PMC9098874; doi:10.1038/s41467-022-30435-z)
Supplement: Supplementary file 2 — Description of Additional Supplementary Files [file 41467_2022_30435_MOESM2_ESM.pdf]

Description of Additional Supplementary Files

File Name: Supplementary Movie 1
Description: Multijet forming process of 3D reaction electrospinning.

File Name: Supplementary Movie 2
Description: Stretching process of ICCA.

File Name: Supplementary Movie 3
Description: Stretching process of crimped ceramic nanofiber.
